# Exploring the Intersectionality of Place and Gender Among Older Adults in Ghana: An Examination of Women’s Disability Disadvantage

**DOI:** 10.1093/geroni/igad134

**Published:** 2023-12-19

**Authors:** Shane D Burns, Latrica E Best, Solomon Amoatey

**Affiliations:** Population Studies Center, University of Michigan, Ann Arbor, Michigan, USA; Sociology Department and African and African Diaspora Studies Program, Boston College, Chesnut Hill, Massachusetts, USA; Department of Sociology, University of Louisville, Louisville, Kentucky, USA

**Keywords:** Activities of daily living, Morbidities, Rural, Urban, Widowhood

## Abstract

**Background and Objectives:**

Ghana’s older adult population is growing rapidly and is projected to double by 2050. It is well-documented that social, health, and housing factors influence segmented aging trajectories that lead to disparate rates of disability. However, little is known about how the intersection of place (i.e., urban and rural) and gender (i.e., woman and man) inform rates of disability among older Ghanaians. We seek to examine this gap in the literature through an intersectional approach.

**Research Design and Methods:**

Using logistic regression with Wave 1 (2007/2008) data from the World Health Organization’s Study on global AGEing and adult health (SAGE) Ghana, we investigate the prevalence of reporting activities of daily living (ADL) disability among respondents ages 50+ (*n* = 4,106). To document gender differences by place, we compute separate adjusted odds ratio models among urban and rural respondents. We also control for health, social, and housing factors that might explain gender differences.

**Results:**

Compared to urban men, urban women’s ADL disability disadvantage was explained by marital status, particularly widowhood. In contrast, rural women consistently reported an ADL disability disadvantage when compared to rural men. Additionally, we found that the morbidity profiles of those who reported ADL disability differed by place and that certain ADL difficulties (i.e., bed transferring and toileting) were especially common among women respondents.

**Discussion and Implications:**

Women, regardless of urban or rural residence, were especially vulnerable to ADL disability. Marital status, particularly widows, explained the difference in disability risk between urban men and urban women. This finding suggests that urban women’s risk of ADL disability is attenuated during the partnership. Also, we speculate that varied morbidity associations with ADL disability are due to different stressors in urban versus rural environments. These findings also generate further interest in about rural women’s disability disadvantage.

**Translational Significance:** Place and gender are often attributed to disability disparities, though there is limited research on the intersectionality of these concepts, particularly in a sub-Saharan African context. We found that the urban gender difference in ADL disability was explained by marital status, particularly widowhood. Our intersectional examination of the disablement process in Ghana can help inform future research, policy, and programming related to the health of older adults in sub-Saharan Africa.

Disability is a key concern within aging research due to its relationship with poor health and limited social ties (Lestari et al., 2019). Existing research shows that older adult risk of disability is especially high among rural residents and women (Hosseinpoor et al., 2016; Stewart Williams et al., 2015), two groups that regularly experience socioeconomic disadvantage. An intersectional examination of these social categories suggests that the interplay of place (i.e., urban residence and rural residence) and gender (i.e., woman and man) influence segmented disability outcomes. Though there is currently limited health research on the intersection of these concepts (Mwangi & Constance-Huggins, 2019; Roy et al., 2020), especially in a sub-Saharan African context where populations are aging rapidly (Kalu et al., 2021).

The disablement process suggests that social and environmental factors influence functional limitations that limit participation in activities and social life (Verbrugge & Jette, 1994). This is especially pertinent to place since rural and urban settings often display varied infrastructure, lifestyles, and environmental stressors that can shape health differently throughout the life course. Research shows that rural older adults generally have lower education, less access to health care, and higher rates of activities of daily living (ADL) disability than their urban counterparts (Dong & Simon, 2010; Zhang et al., 2017). In addition, housing conditions (e.g., housing materials and plumbing) are known to be associated with reports of physical disability (Clarke & George, 2005; Pinilla-Roncancio et al., 2020). However, results from the World Health Organization’s Study on global AGEing and adult health (SAGE) Ghana Wave 1: The Ghana National Report found very little difference in the prevalence of ADL disability between older urban (41.8%) and rural (41.6%) residents (Biritwum et al., 2013). Very little is known about how gender is situated within urban and rural disability among older adults in Ghana, one of sub-Saharan Africa’s fastest-aging countries (He et al., 2020).

Gender contributes to the disablement process because men and women often have varying biological and social experiences that shape their health throughout the life course. For example, post-menopausal women are notably more at risk than men of developing arthritis, a morbidity commonly associated with disability (Hughes & Dunlop, 1995). Women in sub-Saharan Africa are regularly subjected to gender-based social divisions. Early in life, girls and young women face barriers to earning an education. This educational disadvantage influences high rates of illiteracy and precarious employment among women (Arbache et al., 2010). Also, women are oftentimes pressured to prioritize domestic chores that are associated with being a homemaker, mother, and/or wife. In turn, these patriarchal conventions result in women facing socioeconomic disadvantage compared to men, thus contributing to women’s disability disadvantage later in life (Zhong et al., 2017). Also, widowhood, which is more common among older women than older men, is associated with a particularly high risk of disability (Ferraro, 1986; Goldman et al., 1995; Wandera et al., 2014). Older women’s disability disadvantage is consistently observed across the world (Hosseinpoor et al., 2016), even in West African countries such as Burkina Faso, Mali, and Senegal (Miszkurka et al., 2012). Specifically, the Ghana National report showed that half (50.1%) of older women reported ADL disability, compared to just over a third (34.1%) of older men (Biritwum et al., 2013).

Despite these findings in the literature, the presence of older adult disability at the intersection of place and gender is largely unexplored. One study using SAGE India data investigated how regional variations of gender-based education inequities influenced reports of functional limitation (Roy et al., 2020). They found that the influence of education on functional limitation varied by region but only for women. In addition, they observed that some less socioeconomically advanced regions of India reported comparatively low rates of functional limitation. Such research has not been explored in a sub-Saharan African context, as urban–rural distinctions in older adult health research are limited (Kalu et al., 2021). Previous research using SAGE Ghana data has found that women report greater rates of ADL disability than men (Amegbor et al., 2018), especially those with multiple chronic conditions (Darkwah et al., 2022). Older Ghanaian women also report greater care needs than their men counterparts (Awuviry-Newton et al., 2022a, 2022b). Gender norms in Ghana oftentimes restrict older women to household spaces where they are ascribed to domestic roles (Braimah & Rosenberg, 2021); also, older Ghanaian women report discrimination and abuse more often than their men counterparts. Using intersectionality as a guiding theoretical framework, we add to this literature by addressing the following question: What factors contribute to the unique gender difference in ADL disability in urban versus rural settings?

Intersectionality is a useful theoretical framework for examining the disablement process by gender and place. Anchored in United States-based Black feminist scholarship (Crenshaw, 1989), intersectionality emerged as a theoretical and social inquiry into understanding the ways in which Black women, due to their race and gender, were “doubly disadvantaged” and thus more vulnerable to sexual and class oppression (Thomas Tobin et al., 2023). Intersectionality has evolved into a theoretical framework and analytical tool to examine how multiple, interlocking systems of oppression, or matrix of domination (Collins, 2019), intersect to both situate and, in many cases, constrict people based on their intersecting social identities (Thomas Tobin et al., 2023). Intersectionality is not merely concerned with intersecting social identities; it addresses issues related to power, social inequality, relationality, social context, complexity, and social justice (Collins, 2019). Intersectionality has grown into a useful framework beyond analyses of race, class, and gender, providing critical insight into the ways other social identities, such as disability (Artiles, 2013), age (Thomas Tobin et al., 2023), and nation (Purkayastha, 2012), “operate not as unitary, mutually exclusive entities, but as reciprocally constructing phenomena that in turn shape complex social inequalities” (Collins, 2015, p. 2).

Methodologically, early intersectional health disparities research was largely qualitative (Bauer, 2014), given the usefulness of qualitative methodology to capture and examine complex, socially constructed phenomena (Misra et al., 2021). However, there have been increasing efforts to explore how to best incorporate key tenets of intersectionality within quantitative health disparities research (Bauer, 2014; Harari & Lee, 2021). Additionally, U.S. government agencies, such as the National Institutes of Health, have recently championed more public and population health research and grant applications focused on intersectional approaches to health (Alvidrez et al., 2021). There are many potential approaches to incorporating intersectionality into health disparities research. Bauer (2014) offers three ways in which intersectionality can inform quantitative approaches to population health inequities. First, intersectionality can inform descriptive health disparities research that can be used to further identify, measure, and evaluate health inequalities across time and space. Second, intersectionality can be utilized within statistical analyses to identify and examine multilevel, and intersecting, causes of health inequalities (Evans et al., 2018). Finally, intersectionality’s key tenets, such as social justice, can be beneficial in establishing important health and public policy-related interventions at the individual and population level as well as providing perspective on the ways such strategies can intersect (Bauer, 2014).

Using an intersectionality framework to explore reports of ADL disability by place and gender among older Ghanaians allows us to add necessary context to the literature on disability and aging in Ghana. Although intersectionality has been utilized within feminist scholarship in non-Western countries (Gouws, 2017; Kapilashrami & Hankivsky, 2018), research using intersectionality as its theoretical foundation remains largely untapped, particularly within sub-Saharan Africa (Meer & Müller, 2017; Rutagumirwa & Bailey, 2022). Also, scholarship highlighting intersectional, age-related concerns is equally limited. Similarly, intersectional approaches to disability are well-documented within disability and gender studies, especially within Western contexts (Naples et al., 2019). Whereas Western researchers have utilized intersectionality as a framework in which to interrogate both the whitewashing of disability studies and the ways in which disability intersects with other social identities to create or sustain inequalities, scholars examining intersectionality and disability in non-Western countries are also concerned with its applicability outside of Western societies, where social identities and processes differ (Stienstra & Nyerere, 2016).

Thus, we seek to address this gap in the gerontological literature by taking an intersectional approach to investigate reports of ADL disability by place and gender among older adults in Ghana. We separately examine these gender differences in ADL disability by place, with special attention to demographic, health, and housing indicators. In addition, we investigate gender differences in ADL disability by administrative region as well as explore individual ADL difficulty gender differences by place. By doing so, we expand upon previous literature that argues for the inclusion of location as a crucial axis within intersectional analyses of health disparities (Mwangi & Constance-Huggins, 2019; Roy et al., 2020). Past research also argues for the use of geographical location within intersectionality research that goes beyond global and national understandings of place to capture more nuanced perspectives of how social identities and processes interact within and between communities with unequally distributed resources and opportunities (Roy et al., 2020; Whittle & Inhorn, 2001). Such research highlights disability within the understudied intersection of place and gender while addressing the importance of rapid aging in sub-Saharan Africa, namely Ghana.

In 2021, Ghana had a population of approximately 30.8 million, with 12.3% being aged 50+ (women: 13.1%; men: 11.4%; Ghana Statistical Service, 2023). Also, the proportion of rural Ghanaians aged 50+ (total: 12.8%; women: 13.8%; men: 11.8%) was slightly higher than urban Ghanaians aged 50+ (total: 11.7%; women: 12.6%; and men: 10.9%). By 2050, it is projected that 20.5% of Ghanaians will be aged 50+ (women: 21.9%; men: 19.2%), a roughly 60% increase in just 30 years (United States Census Bureau, 2023). Ghana’s Persons with Disability Act, 2006 (Act 715) was passed by the Ghanaian Parliament to address thematic provisions for people with disabilities: rights, accessibility, education, and employment (Asante & Sasu, 2015). In 2010, the Government of Ghana introduced the National Aging Policy (NAP) with the goal of integrating older adults into mainstream society through efforts that address poverty, health, and living environments (Ashirifi et al., 2022). However, a recent report written by Ghana’s civil society organizations argued that Act 715 “fails to provide” for older adults with disabilities (GFDO & CRPD Technical Committee, 2022, p. 10). In addition, NAP has yet to be funded and implemented (Ashirifi et al., 2022). Therefore, while the Government of Ghana has demonstrated interest in protecting the rights of older adults and people with disabilities, there have seemingly been limited efforts to fulfill these expectations. Highlighting intersectional approaches to disability and aging from a policy perspective can not only shape how policymakers view the needs of Ghanaians but also provide context essential to creating equitable and achievable policies.

## Method

### Data

We used data from Wave 1 (2007/2008) of the World Health Organization’s Study on global AGEing and adult health (SAGE) Ghana, a nationally representative data set that collects comprehensive longitudinal information on the health and well-being of adult populations and the aging process. SAGE Ghana was selected for our analyses as it includes disability, health, and housing information on older adults. SAGE Ghana started with 5,067 respondents. We limited our sample to those ages 50+ (*n* = 4,271). Last, we excluded individuals who were missing reports of the covariates (*n* = 165), leading to an analytical sample of *n* = 4,106.

### Measures

Activities of daily living disability

Respondents reported that they had difficulty in any of the following six separate activities of daily living: bathing, bed transferring, dressing, eating, moving around the home, and toileting. We created a binary variable that describes if respondents reported difficulty in one or more of these six categories. *Place.* Respondents reported if they lived in either an urban (reference) or rural setting. *Gender.* Respondents were asked to report their sex: male or female. We renamed this variable gender and recoded the response options to men (reference) and women.

### Covariates

We also accounted for demographic, socioeconomic, and health characteristics related to disability. The *Age Group* was coded as: 50–59 (reference), 60–69, 70–70, and 80+. *Region* was categorized as: Greater Accra (reference), Ashanti, Brong Ahafo, Central, Eastern, Northern, Upper East, Upper West, Volta, and Western. Due to our usage of 2007/2008 data, we analyzed Ghana’s prior 10 administrative regions which were redrawn into 16 regions after a 2018 referendum (van Gyampo, 2018). *Education* was coded as: “no formal education” (reference) if they reported no education, “less than secondary” if they reported less than primary or completed primary education, and “secondary or more” if they completed secondary education or any subsequent diploma/degree. We include seven individual morbidity binary variables that report the following diagnoses: *Stroke*, *Angina*, *Diabetes*, *Depression*, *Hypertension*, *Lung Condition*, and *Cataract*. *Marital status* was coded as: married/cohabiting (reference), separated/divorced, and widowed; also, the notably small number of those who reported being “never married” (*n* = 46) were coded into the separated/divorced group. *Earth floors* were coded as: hard floor (reference) and earth floor. *Nondurable walls* were coded as: has durable walls (reference) and nondurable walls. *No piped water* was coded as: has pipe water (reference) no piped water.

### Analytic Strategy

We first calculated weighted descriptive statistics for older adults by place and gender. Following, we tested these variables for gender differences among both urban and rural respondents, separately. We then tested for age-adjusted gender differences in reporting ADL disability within each of the 10 administrative regions of Ghana. We then tested for age-adjusted gender differences by reporting all six individual ADL difficulties for both urban and rural respondents, separately. Finally, we conducted adjusted odds ratio analyses, separately for both urban and rural respondents, to test for gender differences in reporting ADL disability. Existing research primarily finds that health conditions explain the gender differences in disability (Murtagh & Hubert, 2004; Zeki Al Hazzouri et al., 2011). For for a more thorough analysis, we also control for social and housing indicators that often differ by place and/or gender. The models were as follows: Model 1 included region, Model 2 added education, and Model 3 incorporated six morbidity variables (i.e., stroke, angina, diabetes, depression, hypertension, lung condition, and cataract). Model 4 employed marital status, and Model 5 included housing condition variables (i.e., earth floors; nondurable walls; nonflushable toilet; no pipe water). All analyses were conducted on Stata, release 17, and were corrected for the complex survey design using sample weights.

## Results

### Sample Characteristics

The sample characteristics by place and gender are shown in Table 1 (*n* = 4,106); also provided in the table are gender differences by place for each covariate. Reports of ADL disability were highest among rural women (54.1%) and urban women (51.8%), followed by rural men (42.2%) and urban men (37.0%). For the age group, urban men had the notably highest proportion of those aged 50–59 (47.2%). Respondents aged 60–69 were similar across the four place/gender groups (range: 26.2%–29.9%). Those aged 70–79 were particularly higher among urban women (24.0%) and rural women (27.7%). Respondents ages 80+ represented just over 10% of all place/gender groups, except urban men (6.7%). Urban men had the greatest proportion of respondents with secondary or more education (51.2%). On the contrary, rural women had the highest proportion of respondents with no education (74.2%). Less than secondary education was mostly similar between all four groups (range: 18.3%–23.6%). For morbidities, reports of having a stroke, diabetes, hypertension, and lung condition were more common among urban respondents. Reports of having angina and depression were more common among women respondents. Also, urban women reported a particularly high rate of having cataracts which is nearly double that other place/gender group. Reports of being married/cohabiting were particularly high among rural men (84.4%) and urban men (82.7%). Both urban women and rural women were more than twice as likely to report being separated/divorced than their men counterparts. Also, reports of being widowed were notably high among urban women (51.4%) and rural women (50.8%). Reports of having earth floors (range: 13.0–14.7%) were mostly similar across the four place/gender groups. Reports of nondurable walls and no piped water were similar between urban men and urban women; however, rural men had slightly higher reports of having nondurable walls and no piped water than rural women.

**Table 1.** Sample Description by Place and Gender (*N* = 4,106)

Variable	Urban			Rural		
	Men *n* = 805		Women *n* = 878	Men *n* = 1,350		Women *n* = 1,073
	*%*	*p* Value	*%*	*%*	*p* Value	*%*
ADL disability	37.0	^***^	51.8	42.2	^***^	54.1
Age group						
50–59	47.2	^***^	38.0	39.1	^*^	35.0
60–69	28.5		27.0	29.9	^*^	26.2
70–79	17.6	^**^	24.0	20.9	^***^	27.7
80+	6.7	^**^	10.9	10.2		11.2
Education						
No education	28.1	^***^	54.7	50.4	^***^	74.2
<Secondary	20.8		21.9	23.6	^**^	18.3
Secondary+	51.2	^***^	23.5	26.0	^***^	7.6
Stroke	3.6		4.3	1.7		1.8
Angina	2.6	^*^	4.7	2.4	^*^	4.2
Diabetes	6.0		7.1	2.0		2.5
Depression	1.2	^*^	2.7	0.7	^**^	2.1
Hypertension	18.3	^***^	27.1	4.7	^***^	10.1
Lung condition	5.0		5.7	3.2		3.1
Cataract	4.5	^**^	8.4	4.7		4.3
Marital status						
Married/cohabiting	82.7	^***^	24.0	84.4	^***^	29.9
Separated/divorced	10.4	^***^	24.6	8.9	^***^	19.3
Widowed	6.8	^***^	51.4	6.7	^***^	50.8
Earth floors	13.5		13.3	14.7		13.0
Nondurable walls	45.5		46.8	53.6	^**^	47.5
No pipe water	56.7		54.7	62.2	^**^	55.7

*Notes:* ADL = activities of daily living. Figures shown are weighted.

Gender difference:

^*^ *p* < .05;

^**^ *p* < .01;

^***^ *p* < .001.

### ADL Disability Gender Differences by Region

**Figure 1.**
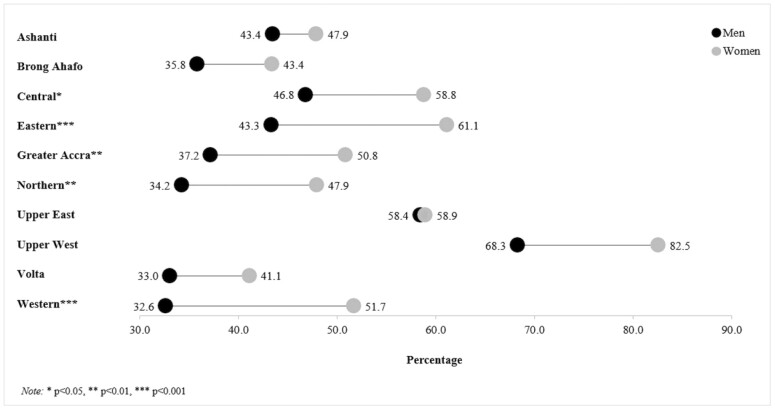
Age-adjusted prevalence of activities of daily living (ADL) disability by gender and region.

We used age-adjusted tests to measure if there were any gender differences in reporting ADL disability within the 10 regions of Ghana (Figure 1). Preliminary tests that separated urban and rural respondents within the 10 regions showed, particularly small subgroups samples, thus we tested all respondents from each region. We found that, compared to the men, the woman counterparts in their respective regions reported significantly greater odds of ADL disability in the following regions: Greater Accra (OR = 1.76; 95% CI: 1.22, 2.55; *p* = .003), Central (OR = 1.67; 95% CI: 1.14, 2.51; *p* = .010), Eastern (OR = 2.22; 95% CI: 1.54, 3.19; *p* = .000), Northern (OR = 1.88; 95% CI: 1.75, 3.01; *p* = .008), and Western (OR = 2.48; 95% CI: 1.67, 3.68; *p* = .000). On the other hand, there were no significant ADL disability gender differences observed in the regions of Ashanti, Brong Ahafo, Upper East, Upper West, and Volta.

### Individual ADL Difficulty Gender Differences by Place

**Figure 2.**
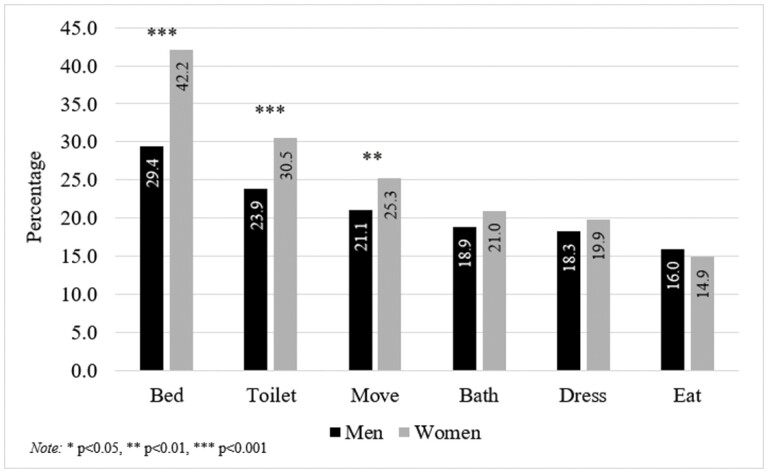
Age-adjusted prevalence of individual activities of daily living (ADL) difficulties by gender—urban respondents.

**Figure 3.**
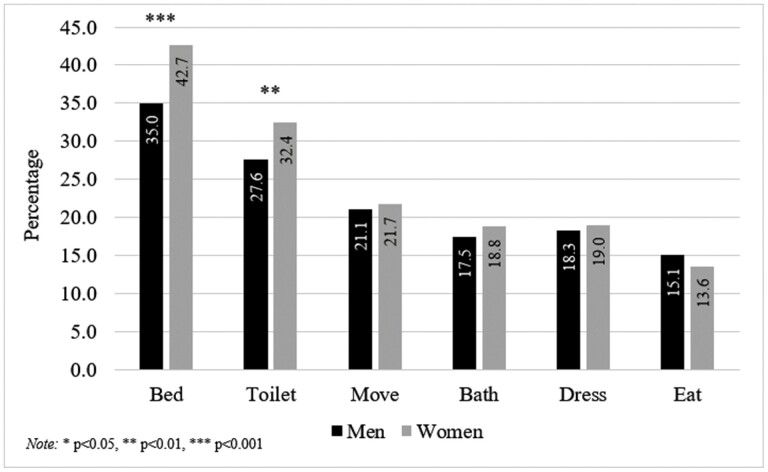
Age-adjusted prevalence of individual activities of daily living (ADL) difficulties by gender—rural respondents.

ADLs (i.e., bathing, bed transferring, dressing, eating, and toileting) each provide their own unique bodily demands and can be influenced by one’s environment (Saito et al., 2014). Thus, we display the age-adjusted prevalence of each ADL difficulty by place and gender then separately test for gender differences among both urban (Figure 2) and rural (Figure 3) respondents. Upon observation, bed transferring was the most reported ADL difficulty across all four place/gender groups and was significantly higher among women in both urban (OR = 1.80; 95% CI: 1.46, 2.21; *p* = .000) and rural (OR = 1.42; 95% CI: 1.20, 1.69; *p* = .000) groups. Toileting was the second most reported ADL difficulty across all four groups and was also significantly higher among women in both urban (OR = 1.42; 95% CI: 1.14, 1.78; *p* = .002) and rural (OR = 1.28; 95% CI: 1.07, 1.53; *p* = .008) groups. Moving around the home was the third most reported ADL difficulty across all four groups but only showed a significant gender difference among urban respondents (OR = 1.28; 95% CI: 1.01, 1.61; *p* = .041). Among both urban and rural respondents, there were no gender differences in reports of bathing, dressing, or eating difficulty.

### Multivariate Analyses

Table 2 (*n* = 1,683) shows adjusted odds ratios of reporting ADL disability among urban respondents. In our initial model, which included region (Model 1), women reported greater odds of ADL disability than men (OR = 1.66; 95% CI: 1.35, 2.04; *p* = .000). Compared to respondents in Greater Accra, significantly higher odds of ADL disability were observed in the following regions: Brong Ahafo (OR = 0.52; 95% CI: .35, .78; *p* = .002), Central (OR = 1.49; 95% CI: 1.02, 2.18; *p* = .040), Upper East (OR = 4.25; 95% CI: 1.89, 9.55; *p* = .000), and Volta (OR = 0.53; 95% CI: .35, .82; *p* = .005). Compared to respondents aged 50–59, significantly higher odds of ADL disability were found in all other age groups: 60–69 (OR = 1.44; 95% CI: 1.13, 1.85; *p* = .004), 70–79 (OR = 3.00; 95% CI: 2.28, 3.94; *p* = .000), and 80+ (OR = 6.44; 95% CI: 4.24, 9.77; *p* = .000). Adding education to our regression (Model 2) did not change women’s greater odds of ADL disability. However, compared to respondents with no education, those with a secondary or more education reported significantly lower odds of ADL disability (OR = 0.70; 95% CI: .54, .92; *p* = .010). Also, adding education to our regression explained the disability disparity among respondents in the Central region. Introducing morbidities to our regression (Model 3) had very little impact on women’s greater odds of reporting ADL disability. However, having a stroke (OR = 3.83; 95% CI: 2.04, 7.20; *p* = .000), depression (OR = 2.38; 95% CI: 1.04, 5.45; *p* = .041), or lung condition (OR = 2.00; 95% CI: 1.24, 3.23; *p* = .005) were associated with higher odds of ADL disability than those who did not report having these morbidities. Adding morbidities to our regression explained the ADL disability risk among respondents aged 60–69 while also showing that respondents in the Central region had significantly higher odds of ADL disability than those in Greater Accra (OR = 1.57; 95% CI: 1.06, 2.34; *p* = .025). The inclusion of marital status (Model 4) explains women’s ADL disability risk. Specifically, compared to respondents who were married/cohabiting, those who were widowed reported significantly greater odds of ADL disability (OR = 1.39; 95% CI: 1.03, 1.88; *p* = .033). Housing condition variables were then added to our regression (Model 5), though it did not provide any ADL disability gender difference. However, compared to respondents with a hard floor, those with an earth floor had significantly higher odds of reporting ADL disability (OR = 1.57; 95% CI: 1.12, 2.22; *p* = .010).

**Table 2.** Adjusted Odds Ratios of Reporting Activities of Daily Living (ADL) Disability—Urban Respondents (*N* = 1,683)

Variable	M1: Age group			M2: Education			M3: Morbidities			M4: Marital status			M5: Housing conditions		
	OR	95% CI		OR	95% CI		OR	95% CI		OR	95% CI		OR	95% CI	
		LL	UL		LL	UL		LL	UL		LL	UL		LL	UL
Women	1.66^***^	1.35	2.04	1.52^***^	1.22	1.89	1.46^**^	1.17	1.82	1.26	0.97	1.65	1.26	0.96	1.65
(50–59)															
60–69	1.44^**^	1.13	1.85	1.37^*^	1.07	1.77	1.23	0.95	1.60	1.19	0.92	1.54	1.18	0.91	1.54
70–79	3.00^***^	2.28	3.94	2.71^***^	2.04	3.61	2.42^***^	1.80	3.24	2.26^***^	1.67	3.05	2.24^***^	1.66	3.03
80+	6.44^***^	4.24	9.77	5.61^***^	3.64	8.65	4.92^***^	3.16	7.67	4.48^***^	2.85	7.03	4.50^***^	2.86	7.07
Greater Accra															
Ashanti	0.89	0.65	1.20	0.83	0.61	1.13	0.88	0.65	1.21	0.89	0.65	1.22	0.96	0.70	1.33
Brong Ahafo	0.52^**^	0.35	0.78	0.49^**^	0.32	0.73	0.53^**^	0.35	0.80	0.53^**^	0.35	0.81	0.57^**^	0.37	0.87
Central	1.49^*^	1.02	2.18	1.38	0.94	2.03	1.57^*^	1.06	2.34	1.56^*^	1.05	2.32	1.68^*^	1.12	2.52
Eastern	1.14	0.79	1.66	1.10	0.76	1.61	1.17	0.80	1.71	1.16	0.79	1.71	1.22	0.83	1.79
Northern	0.86	0.51	1.45	0.72	0.42	1.24	0.84	0.48	1.44	0.84	0.49	1.45	0.91	0.52	1.59
Upper East	4.25^***^	1.89	9.55	3.47^**^	1.53	7.90	4.23^**^	1.85	9.67	4.16^**^	1.82	9.53	4.44^**^	1.91	10.32
Upper West	1.26	0.58	2.77	1.19	0.54	2.64	1.26	0.57	2.80	1.27	0.57	2.82	1.15	0.51	2.61
Volta	0.53^**^	0.35	0.82	0.51^**^	0.33	0.80	0.53^**^	0.34	0.83	0.53^**^	0.34	0.83	0.56^*^	0.36	0.89
Western	1.08	0.73	1.60	1.05	0.70	1.56	1.12	0.74	1.68	1.11	0.74	1.67	1.09	0.72	1.65
(No education)															
<Secondary				0.87	0.65	1.16	0.82	0.61	1.10	0.83	0.62	1.12	0.83	0.62	1.11
Secondary+				0.70^**^	0.54	0.92	0.67^**^	0.51	0.89	0.69^**^	0.52	0.91	0.69^**^	0.52	0.91
Stroke							3.83^***^	2.04	7.20	3.81^***^	2.03	7.15	3.76^***^	2.00	7.07
Angina							1.32	0.75	2.34	1.26	0.71	2.24	1.31	0.74	2.34
Diabetes							1.12	0.72	1.75	1.12	0.72	1.75	1.13	0.72	1.75
Depression							2.38^*^	1.04	5.45	2.38^*^	1.05	5.42	2.45^*^	1.07	5.57
Hypertension							1.20	0.92	1.55	1.19	0.92	1.55	1.20	0.92	1.56
Lung condition							2.00^**^	1.24	3.23	2.03^**^	1.26	3.28	2.04^**^	1.26	3.30
Cataract							1.46	0.94	2.28	1.48	0.95	2.30	1.46	0.94	2.28
(Married/cohabiting)															
Separated/divorced										1.08	0.79	1.48	1.09	0.79	1.49
Widowed										1.39^*^	1.03	1.88	1.39^*^	1.02	1.88
Earth floor													1.57^**^	1.12	2.22
Nondurable walls													0.97	0.75	1.25
No pipe water													1.00	0.78	1.27
*Constant*	0.39	0.30	0.51	0.53	0.37	0.75	0.47	0.32	0.67	0.46	0.32	0.66	0.42	0.29	0.62

*Notes:* OR = odds ratio; CI = confidence interval.

^*^ *p* < .05;

^**^ *p* < .01;

^***^ *p* < .001.

Table 3 (*n* = 2,423) shows adjusted odds ratios of reporting ADL disability among rural respondents. In our initial model, which included region (Model 1), women reported greater odds of ADL disability than men (OR = 1.70; 95% CI: 1.43, 2.03; *p* = .000). Compared to respondents in Greater Accra, odds of ADL disability were significantly higher in the following regions: Upper East (OR = 1.84; 95% CI: 1.01, 3.33; *p* = .046) and Upper West (OR = 6.70; 95% CI: 3.12, 14.12; *p* = .000). Compared to respondents aged 50–59, we found significantly higher odds of ADL disability in all other age groups: 60–69 (OR = 1.86; 95% CI: 1.50, 2.30; *p* = .000), 70–79 (OR = 3.46; 95% CI: 2.76, 4.34; *p* = .000), and 80+ (OR = 6.56; 95% CI: 4.76, 9.04; *p* = .000). Adding education to our regression (Model 2) did not change women’s greater odds of ADL disability, nor were there any significantly different reports of ADL disability between education groups. Introducing morbidities to our regression (Model 3) had very little impact on women’s greater odds of reporting ADL disability. However, reports of having a stroke (OR = 4.54; 95% CI: 2.02, 10.23; *p* = .000), hypertension (OR = 1.65; 95% CI: 1.16, 2.35; *p* = .005), lung condition (OR = 1.87; 95% CI: 1.12, 3.11; *p* = .017), or cataracts (OR = 1.94; 95% CI: 1.23, 3.06; *p* = .004) were associated with higher odds of ADL disability than those who did not report having these morbidities. The inclusion of marital status (Model 4) did not explain women’s ADL disability risk. However, compared to respondents who were married/cohabiting, those who were widowed reported significantly greater odds of ADL disability (OR = 1.34; 95% CI: 1.04, 1.72; *p* = .024). Housing condition variables were then added to our regression (Model 5), though it did not explain any ADL disability gender difference. In addition, none of the housing condition variables were associated with odds of ADL disability.

**Table 3.** Adjusted Odds Ratios of Reporting Activities of Daily Living (ADL) Disability—Rural Respondents (*N* = 2,423)

Variable	M1: Age group			M2: Education			M3: Morbidities			M4: Marital status			M5: Housing conditions		
	OR	95% CI		OR	95% CI		OR	95% CI		OR	95% CI		OR	95% CI	
		LL	UL		LL	UL		LL	UL		LL	UL		LL	UL
Women	1.70^***^	1.43	2.03	1.67^***^	1.38	2.02	1.65^***^	1.36	2.00	1.44^**^	1.15	1.80	1.45^**^	1.15	1.81
(50–59)															
60–69	1.86^***^	1.50	2.30	1.85^***^	1.50	2.29	1.77^***^	1.42	2.19	1.71^***^	1.38	2.13	1.72^***^	1.38	2.14
70–79	3.46^***^	2.76	4.34	3.45^***^	2.73	4.35	3.13^***^	2.47	3.97	2.96^***^	2.32	3.77	2.96^***^	2.33	3.78
80+	6.56^***^	4.76	9.04	6.51^***^	4.67	9.08	6.12^***^	4.38	8.57	5.63^***^	3.99	7.95	5.63^***^	3.99	7.95
(Greater Accra)															
Ashanti	1.26	0.71	2.24	1.27	0.72	2.26	1.34	0.75	2.41	1.40	0.78	2.51	1.39	0.77	2.49
Brong Ahafo	0.91	0.50	1.63	0.91	0.50	1.63	0.98	0.54	1.77	1.02	0.56	1.86	1.03	0.57	1.87
Central	1.31	0.73	2.34	1.31	0.74	2.35	1.37	0.76	2.47	1.43	0.79	2.59	1.44	0.80	2.60
Eastern	1.39	0.79	2.46	1.39	0.79	2.45	1.45	0.82	2.58	1.53	0.86	2.72	1.48	0.83	2.65
Northern	0.81	0.45	1.44	0.81	0.45	1.45	0.87	0.48	1.57	0.93	0.51	1.68	0.91	0.50	1.65
Upper East	1.84^*^	1.01	3.33	1.84^*^	1.01	3.35	2.08^*^	1.13	3.81	2.21^*^	1.20	4.08	2.14^*^	1.15	3.98
Upper West	6.70^***^	3.18	14.12	6.73^***^	3.17	14.26	7.39^***^	3.46	15.77	7.80^***^	3.65	16.69	7.49^***^	3.48	16.11
Volta	0.68	0.38	1.22	0.68	0.38	1.22	0.70	0.39	1.28	0.73	0.40	1.33	0.75	0.41	1.36
Western	0.85	0.48	1.51	0.88	0.49	1.55	0.93	0.52	1.66	0.96	0.54	1.72	0.94	0.53	1.69
(No education)															
<Secondary				1.10	0.87	1.39	1.05	0.83	1.34	1.06	0.83	1.35	1.06	0.83	1.35
Secondary+				0.89	0.68	1.17	0.82	0.62	1.08	0.82	0.62	1.08	0.82	0.62	1.08
Stroke							4.54^***^	2.02	10.23	4.54^***^	2.02	10.20	4.52^***^	2.01	10.18
Angina							0.76	0.46	1.26	0.76	0.46	1.25	0.75	0.46	1.25
Diabetes							1.24	0.69	2.25	1.27	0.70	2.30	1.25	0.69	2.28
Depression							1.08	0.47	2.47	1.08	0.47	2.49	1.08	0.47	2.47
Hypertension							1.65^**^	1.16	2.35	1.67^**^	1.17	2.38	1.68^**^	1.18	2.39
Lung condition							1.87^*^	1.12	3.11	1.82^*^	1.09	3.04	1.83^*^	1.10	3.05
Cataract							1.94^**^	1.23	3.06	1.95^**^	1.24	3.07	1.95^**^	1.24	3.07
(Married/cohabiting)															
Separated/divorced										1.19	0.90	1.56	1.18	0.90	1.56
Widowed										1.34^*^	1.04	1.72	1.33^*^	1.04	1.72
Earth floor													1.05	0.80	1.39
Nondurable walls													0.99	0.80	1.22
No pipe water													1.11	0.91	1.35
*Constant*	0.32	0.19	0.55	0.32	0.18	0.56	0.29	0.17	0.52	0.28	0.16	0.49	0.26	0.15	0.47

*Notes:* OR = odds ratio; CI = confidence interval.

^*^ *p* < .05;

^**^ *p* < .01;

^***^ *p* < .001.

## Discussion

The purpose of this study was to determine gender differences in reports of ADL disability among urban and rural-dwelling older adults in Ghana. This is the first known study to separately examine urban and rural gender disparities in ADL disability in a sub-Saharan African context. Our study addresses indicators of urban women’s disability disadvantage while also dissecting gender differences among individual ADL difficulties and Ghana’s regions. We contribute to the growing body of literature that addresses aging in sub-Saharan Africa by incorporating an intersectional analysis of older adult disability in Ghana.

Our primary finding was that urban women’s disability disadvantage could be explained by marital status. This finding supplements previous studies that suggest marital status, particularly widowhood, is associated with a greater risk of disability than those who are married (Ferraro, 1986; Goldman et al., 1995), particularly in a sub-Saharan African setting (Wandera et al., 2014). It is documented that rural widows have more contact with their support network than urban widows (Matthews, 1988). As such, widowhood in an urban setting might be especially isolating, and thus contribute, to difficulty performing ADLs. Urban widowhood and living alone, particularly among women, is increasing globally (Sarkar et al., 2023). Also, urban widowed women generally have less retirement income than their men counterparts. As demonstrated, the intersectional complexity of urban residence, womanhood, older adulthood, and disability provide unique challenges to “aging in place” (Rogers et al., 2020). Therefore, it is imperative that Ghana’s civic and governmental bodies promote long-term care (LTC) service provisions (Awuviry-Newton et al., 2022a, 2022b) to hamper the social and economic vulnerabilities that often accompany widowhood.

To develop a more comprehensive understanding of the bidirectional relationship between health and disability, we treated morbidities as individual binary variables. Among urban respondents, we found that a history of a stroke, depression, or lung condition was associated with high odds of ADL disability. Rural respondents also showed a relationship with reports of a stroke and lung condition with ADL disability, in addition to a history of hypertension and cataracts. Indeed, experiencing a stroke can increase the risk of physical disability (Amegbor et al., 2018). As for lung conditions being associated with ADL disability, we speculate that poor air quality in Ghana can debilitate respiratory functioning (Mudu, 2021) and thus inhibit participation in ADLs. There is a known relationship between depression and disability (Kohler et al., 2022); however, urban versus rural differences in depression remain heterogenous and context-specific (Purtle et al., 2019; Yan et al., 2021). Though Ghana’s small group of psychiatric facilities, which are mainly concentrated in its southern regions (Read et al., 2009), might help explain our observed association. Research shows a link between vision loss and mobility (Boakye et al., 2023), though we only observed this among rural respondents. Agricultural occupations often present ocular hazards (e.g., chemicals and ultraviolet sunlight) that can influence poor vision (Boadi-Kusi et al., 2014); this is particularly salient for rural populations who regularly work outdoors with no eye protection. Hypertension is commonly associated with disability (Amegbor et al., 2018) and poor quality of life (Otieno et al., 2022); though we speculate our study’s association between hypertension and rural ADL disability is due to the pronounced lack of health care in rural Ghana (Amoah-Nuamah et al., 2023).

Our regional analysis indicated that there was a gender difference in reporting ADL disability in five of Ghana’s former 10 administrative regions: Greater Accra, Central, Eastern, Northern, and Western. Finding that women reported more ADL disability than men was generally expected. Therefore, the lack of gender differences in specific regions was of particular interest. Upper East and Upper West regions have some of Ghana’s highest rates of income inequality (Ghana Statistical Service, 2015), thus we speculate that the ubiquity of poverty in these regions influences homogenous disability outcomes between men and women. Conversely, we speculate that low levels of poverty inequality in Ashanti contribute to the region’s lack of gender difference in ADL disability. Brong Ahafo and Volta have higher variations of income inequality than the Upper West, Upper East, and Ashanti, thus making it more difficult to posit why men and women report similar rates of ADL disability. We also tested for gender differences in Brong Ahafo and Volta by separately investigating urban and rural respondents, though no differences were observed. However, it is documented that the city of Sunyani (Brong Ahafo) and the region of Adaklu (Volta) have some of the highest rates of income inequality in the country (Ghana Statistical Service, 2015).

Dichotomized analyses of ADL disability are susceptible to overlooking the context of specific ADL difficulties. Thus, we also investigated gender differences in reporting difficulty among each of the six individual ADLs (i.e., bathing, bed transferring, dressing, eating, moving around the home, and toileting). Both urban and rural respondents showed that women reported greater odds of difficulty with bed transferring and toileting. Bed transferring is speculatively a universal (i.e., urban and rural) disadvantage among women due to them having considerably less upper body strength than men (Miller et al., 1993). Also, toileting difficulty, which has been found to be associated with toilet facility type and location (Awuviry-Newton et al., 2023), is likely a universal disadvantage for women due to their need to commonly sit or squat during toilet use. However, we observed that urban women are especially at risk of having difficulty moving around the home. In response, further inquiry on urban women’s mobility disadvantage, such as additional housing conditions and LTC needs (Awuviry-Newton et al., 2022a, 2022b), should be explored.

### Strengths and Limitations

This study provides insight into the health of older Ghanaians by using an intersectional perspective to examine gender differences in disability among urban and rural respondents in a nationally representative sample. To further dissect the disablement process (Verbrugge & Jette, 1994) in a Ghanaian context, we analyzed gender differences in individual ADL difficulties, by administrative region, and highlighted individual morbidity relationships with ADL disability by place. However, this study is not without its limitations. First, we used Wave 1 (2007/2008) data, as it was the only publicly available wave from SAGE Ghana that had both individual and household-level characteristics that were theoretically important for our analysis. While it would be ideal to use the newest wave of SAGE Ghana data (i.e., Wave 3), Wave 2 (2014/2015) household-level data, which is crucial to our theoretical argument, and the entirety of Wave 3 (2019) data were not publicly available during the time of our analysis. As mentioned earlier, this is also why we analyzed Ghana’s prior 10 administrative regions which were redrawn into 16 regions after a 2018 referendum (van Gyampo, 2018). Second, we did not include the occupational variable due to its high number of missing responses. Occupation is an integral component of the disablement process because occupations with physical work demands (i.e., agricultural; industrial) can make people more susceptible to having a functional limitation (Pebley et al., 2021) that leads to disability. Third, interpretations of ADL difficulties vary considerably across populations; for example, toileting limitations might vary between respondents who use a squat toilet versus a toilet chair, while those reporting an eating limitation might use different methods (e.g., cutlery and hand-to-mouth; Saito et al., 2014). Therefore, we cannot ascertain how ADLs are interpreted by each respondent, especially since place and gender can influence varied constructions of these activities.

### Future Directions

This study’s findings underscore the importance of utilizing an intersectional perspective in examining gender differences in disability among urban and rural respondents in Ghana. Though results indicate women’s disability disadvantage, our intersectional analysis shows that this disadvantage varies by marital status and place. Moreover, specific ADL difficulties were particularly common among women. Examining the intersection between gender, place, and disability allows for a more nuanced analysis that highlights key intra- and inter-group distinctions between these intersecting axes. The results also speak to the importance of place beyond national discussions of gender differences in disability. We not only examine rural–urban distinctions in disability but also provide disability profiles by administrative region, highlighting the multidimensional perspectives often championed in studies examining the inclusion of intersectionality within quantitative health disparities research.

Future research on this topic would benefit from investigating gender differences within Ghana’s new 16 administrative region system which more accurately represents the population and infrastructural diversity of Ghana. This recommendation is particularly salient for the former Brong Ahafo region which is now three separate regions (i.e., Ahafo; Bono East; Brong Ahafo), the former Northern region which is now three separate regions (i.e., North East; Northern; and Savannah), the former Volta region which is now two separate regions (i.e., Otia and Volta), and the former Western region which is now two separate regions (i.e., Western and Western North). Conducting research within the context of these newly defined regions could highlight potentially obscured place-based gender disparities in older adult disability.

Future research should incorporate deeper analyses of the disablement process as well as other intersecting social identities. The inclusion of additional waves of SAGE Ghana would allow us to capture disability incidence and changes in disability over time, thus providing an opportunity to better understand the disablement process by gender and place. Examining multiple waves of data allows us to move beyond describing group differences to better understand how social positions shape disability disadvantages across the life course. Also, Ghana’s 2021 Population and Housing Census is a fruitful source of disability and population information (Ghana Statistical Service, 2023), though it does not provide morbidity data; therefore, linkage between demographic and health information can enable analysis of more contemporary data on older adults in Ghana. Additionally, future research should also explore different measures of disability, as past research has shown that, in addition to physical impairment, vision, and hearing impairments contribute to the most common types of disability in Ghana (Tuakli-Wosornu & Haig, 2014).

Finally, our findings also emphasize the importance of exploring other key tenets of intersectionality, such as power and social justice, within disability and aging research and policy in Ghana. This study underscores the need for nationwide LTC and health care reform. Crenshaw’s (1989) genesis of intersectionality was inspired by U.S. law’s separate treatment of race and gender discrimination; thus, we find it particularly important for Ghana’s Persons with Disability Act 2006 to include provisions that mention the rights of women and older adults with disabilities, a sentiment that has been similarly expressed in previous literature (Ocran, 2019). From a theoretical perspective, more research is needed on how intersectionality may vary within the sub-Saharan African context. Historically, intersectionality has traveled unevenly across scholarly arenas (Collins 2015), where researchers in specific disciplines have favored examining some core intersectional tenets, such as social inequality, over others (i.e., social justice). As intersectionality research in general, and disability and aging scholarship specifically, increases in sub-Saharan Africa, its usage may depart from previous studies. Also, the intersectionality framework was established within the United States’s context of race and racialization which may potentially affect how it is applied outside of the United States. Moreover, past research on intersectionality has predominantly focused on race and racialization; future research should explore whether ethnicity or other social identities are important factors in intersectional research on disability. To that end, additional research comparing the intersection of gender, place, and disability with data from SAGE South Africa is imperative to understanding disability profiles in sub-Saharan Africa.

## Conclusion

We found that marital status, particularly widowhood, helped explain urban women’s disability disadvantage. Additionally, we found that the morbidity profiles of those who reported ADL disability differed by place and that certain ADL difficulties (i.e., bed transferring and toileting) were especially common among women respondents. Our study contributes to the growing literature that addresses aging in sub-Saharan Africa by employing an intersectional approach to investigate disability disparities by place and gender. Such research is essential to understanding the intricacies of the disablement process in a rapidly aging, sub-Saharan African setting. Future research on this topic would benefit from studying occupational, health care, and caregiving differences that might shed light on rural women’s disability disadvantage.
